# Force Sensor Based Tool Condition Monitoring Using a Heterogeneous Ensemble Learning Model

**DOI:** 10.3390/s141121588

**Published:** 2014-11-14

**Authors:** Guofeng Wang, Yinwei Yang, Zhimeng Li

**Affiliations:** Key Laboratory of Mechanism Theory and Equipment Design of Ministry of Education, Tianjin University, Tianjin 300072, China; E-Mails: yang.yinwei@163.com (Y.Y.); lzmcxg@163.com (Z.L.)

**Keywords:** heterogeneous ensemble learning, tool condition monitoring, stacking, force sensor

## Abstract

Tool condition monitoring (TCM) plays an important role in improving machining efficiency and guaranteeing workpiece quality. In order to realize reliable recognition of the tool condition, a robust classifier needs to be constructed to depict the relationship between tool wear states and sensory information. However, because of the complexity of the machining process and the uncertainty of the tool wear evolution, it is hard for a single classifier to fit all the collected samples without sacrificing generalization ability. In this paper, heterogeneous ensemble learning is proposed to realize tool condition monitoring in which the support vector machine (SVM), hidden Markov model (HMM) and radius basis function (RBF) are selected as base classifiers and a stacking ensemble strategy is further used to reflect the relationship between the outputs of these base classifiers and tool wear states. Based on the heterogeneous ensemble learning classifier, an online monitoring system is constructed in which the harmonic features are extracted from force signals and a minimal redundancy and maximal relevance (mRMR) algorithm is utilized to select the most prominent features. To verify the effectiveness of the proposed method, a titanium alloy milling experiment was carried out and samples with different tool wear states were collected to build the proposed heterogeneous ensemble learning classifier. Moreover, the homogeneous ensemble learning model and majority voting strategy are also adopted to make a comparison. The analysis and comparison results show that the proposed heterogeneous ensemble learning classifier performs better in both classification accuracy and stability.

## 1. Introduction

Milling is widely used for machining many important parts, such as aviation engine blades, turbine disks, *etc.* Because of the intermittency of the cutting process and poor machinability of the material, tool wear severely during the machining process, which will cause deterioration of workpiece quality and a decrease of machining efficiency. In order to recognize the tool wear states during the milling process, online monitoring systems in which dynamic signals are collected and a classifier is constructed to depict the relationship between the tool wear states and sensory information are preferred. Currently, many models such as support vector machine (SVM) [1,2], artificial neural networks (ANN) [3–6], conditional random field (CRF) [7], hidden Markov model (HMM) [8,9], *etc.* have been proposed to recognize tool wear states. Sun *et al.* [1] used a revised support vector machine (SVM) approach to carry out multi-classification of the tool states. Moreover, a new performance evaluation function was presented by considering manufacturing losses. The experimental results show that the proposed method can reliably perform multi-classification of the tool flank wear and reduce potential manufacturing losses. Shi and Gindy [2] presented a new tool wear predictive model by combining least squares support vector machines (LS-SVM) with the principal component analysis (PCA) method. The effectiveness of the proposed model is demonstrated by experimental results from broaching. Ozel and Nadgir [3] built a system in which a predictive machining approach is combined with a back propagation neural network so as to predict flank wear under different cutting conditions. Based on multi-sensor integration, Kuoa and Cohenb [4] developed a tool condition evaluation system by integrating a radial basis function (RBF) network with a fuzzy logic algorithm. The experimental results show that the proposed system can significantly increase the accuracy of the product profile. Choudhury *et al.* [5] realized the prediction of the flank wear by combining an optoelectronic sensor with a multilayered back propagation network. Silva *et al.* [6] presented two kinds of self-adaptive resonance-based neural networks to classify the tool wear state. The authors claim that a reproducible diagnosis of tool wear can be realized accurately. Wang and Feng [7] proposed a linear chain conditional random field (CRF) model and utilized it for online tool condition monitoring. The application results show that the proposed method can accurately depict the relationship between the feature vectors and the tool wear states. Under three different time scales, Atlas *et al.* [8] adopted HMMs to realize the online monitoring of the milling process. The application shows that the HMMs can give accurate wear predictions. Wang *et al.* [9] also utilized HMMs to build a framework for tool wear monitoring in which feature vectors are extracted from vibration signals measured during the turning process. These applications show that classifier-based monitoring methodologies are effective to recognize the tool wear states. However, the above methods are all based on single classifier strategies, that is, only one classifier is utilized to map the feature vectors and the tool wear categories. In real applications, the spatial distribution of these feature vectors are disperse and irregular due to the complexity of the machining process and tool wear morphology. In such case, over-fitting phenomena can easily occur for the single classifier, which will deteriorate the classification accuracy and generalization ability [10].

In this paper, a tool condition monitoring (TCM) system based on a heterogeneous ensemble learning model is proposed. In this system, force signals are utilized to depict the dynamic characteristics of the tool wear process. The reason for selecting force sensors lies in their reliability and robustness. In fact, as a kind of indirect means, force sensors have been adopted in many monitoring applications. Bhattacharyya used force signals to estimate the tool wear value by using time domain averaging and wavelet transformation [11]. Kaya adopted the average cutting force directly as the input of a neural network to predict tool wear states during machining processes [12]. Cui realized tool wear monitoring by using the coefficients of the cutting force as the indicator [13]. Liu and Altinas built a neural network model to predict the flank wear of turning processes by utilizing the ratio of different force signals as the input [14]. In this paper, considering the periodicity of the milling process, harmonic features are extracted from force sensor information. In addition, to simultaneously improve the relevance and reduce redundancy, a minimal redundancy and maximal relevance (mRMR) algorithm is adopted to filter those less prominent harmonics. Based on these selected features, three different models (SVM, HMM and RBF) are selected as the base classifiers considering diversity and accuracy [15]. Moreover, a SVM-based stacking strategy is constructed to realize nonlinear mapping between the base classifier output and real tool wear states [16]. To verify the effectiveness of the proposed system, titanium alloy milling experiments were carried out and samples from different tool wear states were collected to build the proposed heterogeneous ensemble learning classifier. Moreover, a single classifier, homogeneous ensemble learning classifier and heterogeneous ensemble classifier with majority voting were also constructed to make a comparison with the proposed ensemble learning classifier. The results show that the proposed method performs best in both classification accuracy and stability.

The remainder of the paper is organized as follows: in Section 2, a heterogeneous ensemble learning framework is presented. Moreover, the principle of each base classifier and stacking strategy are also discussed in this section. In Section 3, a tool condition monitoring system is constructed based on heterogeneous ensemble learning and milling experiments are carried out to verify the effectiveness of the TCM system. The comparison with other kinds of classifier shows that the proposed method can achieve higher accuracy and stability. Some useful conclusions are given in Section 4.

## 2. Principle of Heterogeneous Ensemble Learning

### 2.1. Structure of Heterogeneous Ensemble Learning

**Figure 1.**
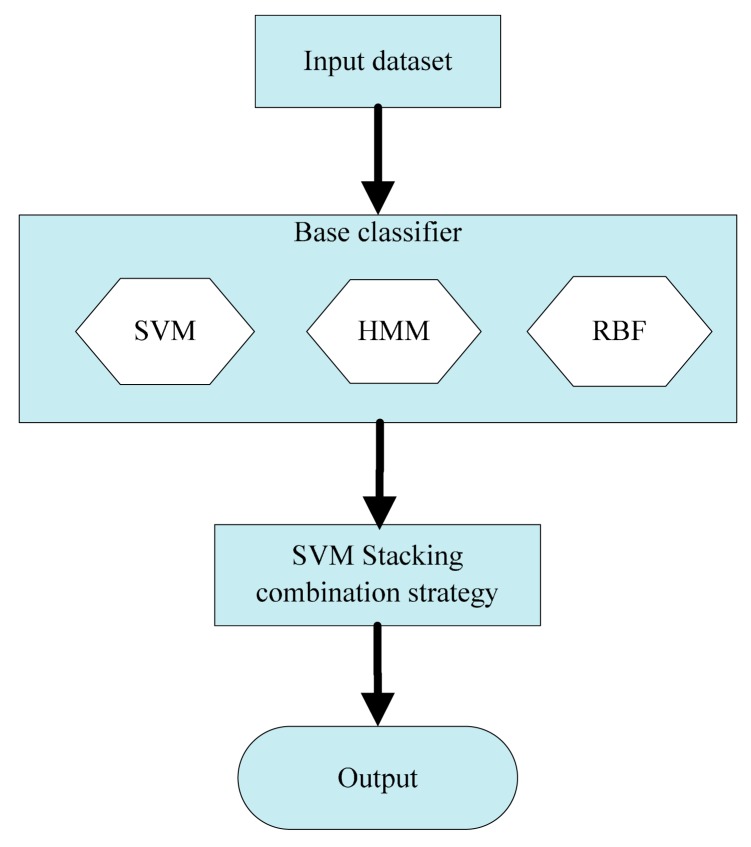
The structure of heterogeneous ensemble learning.

As shown in Figure 1, the heterogeneous ensemble learning model is composed of two parts. The first is the construction of base classifiers. One requirement for these base classifiers is that the accuracy of every single classifier needs to be high enough [15]. The other is the diversity of these base classifiers, which means they should be different from each other. This kind of diversity makes the each base classifier complementary to each other so as to get more accurate decision boundary [17]. In this paper, the SVM, HMM and RBF algorithms are selected as the base classifiers. The SVM algorithm is based on the statistical learning theory, which is trained based on the structural risk minimum principle. In contrast, the RBF network is a multi-layer mapping structure, which is trained based on empirical risk minimization. As for the HMM algorithm, it is a kind of generative model, totally different from the other two classifiers. The second part is the stacking combination strategy in which a meta-learner is used to map the output of the base classifiers to the final tool wear categories. Because the stacking strategy realizes the ensemble of the base classifiers by training a new mapping model, it can greatly improve fault tolerance ability and classification accuracy [18]. Considering the strong nonlinear mapping ability, support vector machine (SVM) is used as a meta-learner to realize stacking combination in the second part.

### 2.2. Principle of Base Classifiers

#### 2.2.1. Support Vector Machine (SVM)

The support vector machine (SVM) is based on the statistical learning theory [19], whose main idea is to transform the samples to a higher dimensional feature space by nonlinear mapping and solve a binary classification problem by selecting the appropriate kernel function in a hyper plane [20]. The main characteristic of a SVM classifier is that it attempts to minimize the structural risk instead of the empirical risk [21]. When the training samples are input, the SVM selects the most important samples, also called support vectors, to realize the maximal margin classification by taking the constraint conditions into consideration. When these samples are not linearly separable, a kernel function needs to be introduced to map the input data into a higher dimensional feature space.

In this paper, Gaussian kernel functions are utilized because they are not sensitive to the outliers and have no equal variance requirement for the input data. The expression of Gaussian function is given as follows:

(1) $$K\left(x_{i},x_{j}\right)=\exp\left(-\gamma {\left\|x_{i}-x_{j}\right\|}^{2}\right)\gamma >0$$

where *K*(*x_i_, x_j_*) is an inner product that maps the input vector *x* ∈ *R^d^* to a high-dimensional space, γ is the variance.

#### 2.2.2. Radial Basis Function (RBF) Network

**Figure 2.**
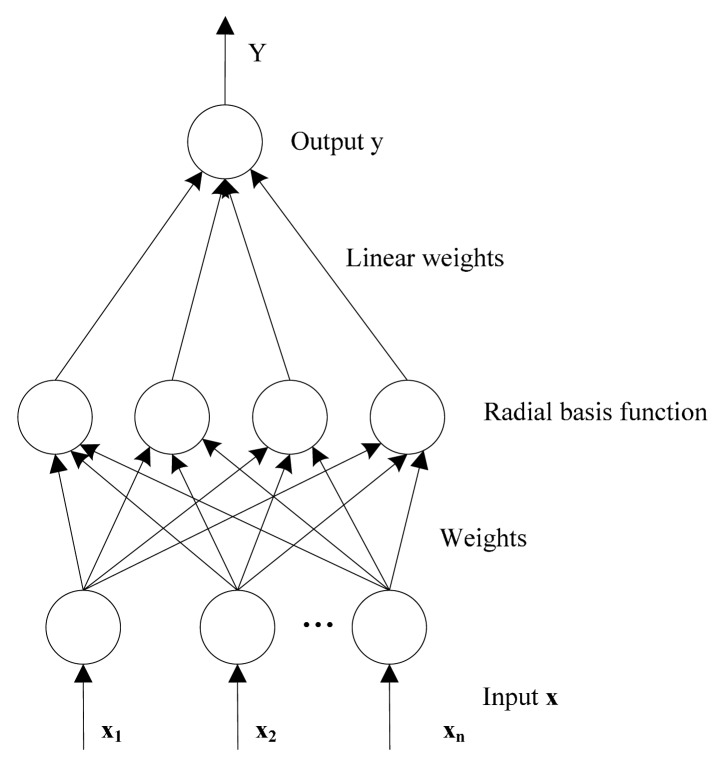
The structure of the RBF network.

The RBF network, which was proposed by Powell [22], is a kind of artificial neural network that uses radial basis functions as the activation function. This network typically has three layers: input layer, hidden layer and linear output layer. The input layer is designed to accept the input data and pass it to the neurons in the hidden layers. The hidden layer consists of a set of radial basis functions by which the Euclidean distance between the center and the network input vector can be calculated [23]. The hidden layer performs a fixed nonlinear transformation and maps the input space onto a new high dimension space. The output layer implements a linear combiner on this new space and the only adjustable parameters are the weights of this linear combiner. The output of the RBF network is shown as follows:

(2) $$y_{k}=w_{0}\sum_{j=1}^{m}w_{jk}\phi (\left\|x=c_{j}\right\|)$$

where ϕ(**·**) is the radial basis function, *w_jk_, j* = (1, 2, …, *m*) and *k* = (1, 2, …, *l*) are the output weights, *w*_0_ is the bias, *x* is a input vector, *c_j_* are the centers associated with the basis function, *m* is the number of hidden neurons, and *l* is the number of classes. The structure of the RBF network is illustrated in Figure 2.

#### 2.2.3. Hidden Markov Model (HMM)

HMM is a kind of generative model-HMM [24], which includes two stochastic processes. One is a Markov process which is used to describe the hidden states transfer sequence. The other is a stochastic process, which is adopted to model the observation sequence of the hidden states. The hidden states transfer sequence is not observable, but can be speculated through the output of the stochastic process. The Markov sequence, which is described by the initial probability distribution vector π with length *N* and state transfer probability matrix *A* with size *N* × *N*. The stochastic process sequence is described by the probability matrix of the observed values *B* whose sizes are equal to *N* × *M*. *M* is the possible number of observed value in each state. Therefore, a HMM model can be described as [7]:

(3) ω={N,M,π,A,B}

There are two steps to use HMM model as a classifier. The first step is the construction of the HMM model. For each tool wear states, the Baum-Welch algorithm is adopted to calculate the model parameters so as to guarantee the maximum probability of the training data. The second is to recognize the tool wear state by inputting the test data into every built model, respectively. The probability of the test data coming from each HMM model is calculated by summing up the probabilities of each hidden state [7] and the final category corresponds to the tool wear state which has the maximum probability.

### 2.3. Stacking Ensemble Strategy

Stacking is a combination strategy in which a meta-learner [25] is constructed to recognize the tool wear category based on the output of different base classifiers. Stack generalization attempts to give an accurate prediction even if the output of a certain base classifier is incorrect. It includes two steps. The first is to organize the prediction of every base classifier into a new dataset. In the second step, a meta-learner is trained based on the dataset and the output is used as the final result. In this paper, SVM is selected as meta-learner to reflect the nonlinear relationship between the output of the base classifier and the final tool wear category.

## 3. Tool Condition Monitoring (TCM) Based on Heterogeneous Ensemble Learning

### 3.1. The Framework of TCM System

**Figure 3.**
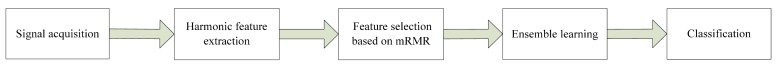
Framework of the tool condition monitoring system.

Based on the proposed ensemble learning classifier, a tool condition monitoring system is constructed, whose structure is shown in Figure 3. The realization of this system is composed of four steps.

The first is signal acquisition. Dynamic signals from force sensor are collected to depict the characteristic of the cutting process. The second is feature extraction. Considering the characteristics of periodic entrance into and exit from the workpiece during the milling process [26], harmonic features are suitable for tool wear monitoring, so they are extracted as the classifier input [27]. The third step is feature selection. Not all amplitudes of the harmonic are sensitive to the variation of the tool wear. Some irrelevant and redundant features can even negatively influence the performance of the model. In this paper, a minimal redundancy and maximal relevance (mRMR) algorithm [28] is used to select the optimal features so as to realize dimension reduction and improve the robustness. The last step is to build a heterogeneous ensemble learning model. In this stage, the SVM, HMM and RBF are selected as base heterogeneous classifiers, and SVM stacking is adopted to integrate the outputs of these base classifiers and judge the final tool wear category.

### 3.2. Feature Extraction and Selection

Harmonic features amplitude of the harmonics in the milling force has been proven to be one of the most effective features to depict the variation of the tool wear during milling processes because of its characteristics of periodic entrance into and exit from the workpiece [27]. For a given cutting force signal *s*(*t*), the amplitude spectrum is given as:

(4) $$p\left(f\right)=\left|S\left(f\right)\right|=\left|\int_{-\infty }^{+\infty }s\left(t\right)e^{-2\pi ft}dt\right|$$

where, *S*(*f*) is the Fourier transform of the original signal *s*(*t*). Based on the cutting speed and cutter geometry, the tooth passing frequency and its harmonics are calculated as:

(5) $$\begin{array}{cc} f_{l}=lVZ/60 & \left(l=1,2,3,\ldots ..\right) \end{array}$$

where, *V* is the rotating speed of the machine tools, *Z* is the number of the cutter and *l* is the order of harmonics. Based on Equations (4) and (5), the amplitude of power spectrum corresponding to different harmonics can be calculated as the candidate feature vectors.

Considering the redundancy of these harmonic features, mRMR feature selection algorithm is further presented to realize dimension reduction. This algorithm is realized by considering the maximum relevance and minimum redundancy criterion simultaneously. The expression is given as follows:

(6) maxΦ(D,R),Φ=D−R

where, *D* is average mutual information and *R* denotes the redundancy of the individual feature vectors. This algorithm is realized by using incremental search strategies and the selection process is terminated if the number of the features meets the requirement. The detailed description of this algorithm is given in [28].

### 3.3. Experimental Setup

**Figure 4.**
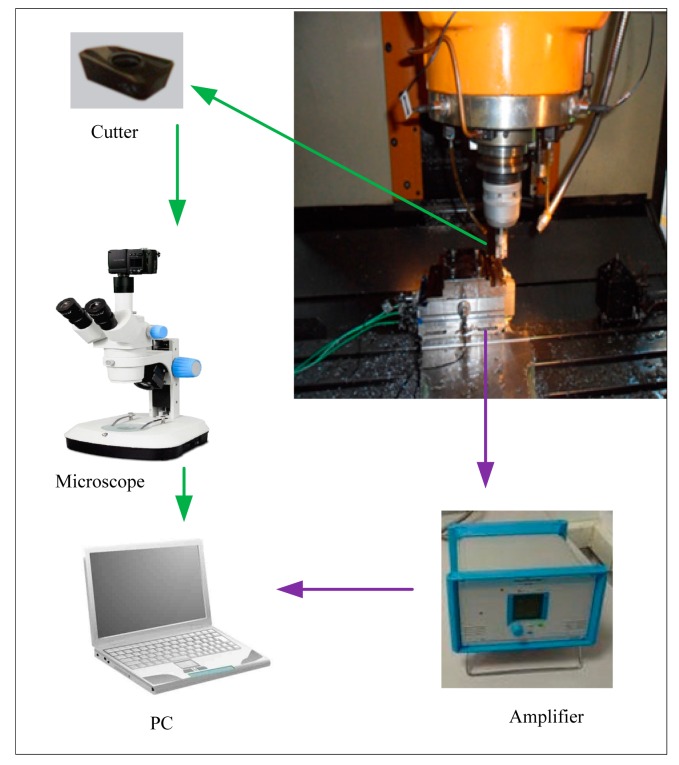
Scheme of tool wear experiment.

In order to verify the effectiveness of this system, titanium alloy milling experiments based on the force sensor were carried out. The structure of the experiment is shown in Figure 4. The experiment was carried out on a FNC-A2 vertical machining center by using the end mill cutter. The cutter is an APMT1604PDER-H2 with three inserts and the tool holder was a DEREK 400R C32-32-200. Besides, the experiment proceeded under lubrication with EP690 half synthetic water soluble cutting fluid. A Kistler 9257 dynamometer was used to collect the force signals in the feeding direction with a sampling rate of 10 K. The cutting parameters used in this experiment are listed in Table 1. The milling of titanium alloy was carried out and the length for each cutting pass was 100 mm. After each pass, the tool wear values on the flank face of all inserts were measured by microscope and their average value was adopted as the indicator of the current tool wear state. As the cutting process proceeded repeatedly, the cutter wore gradually and the experiment was terminated if the tool wear value was larger than 0.35 mm because the cutter was viewed as broken in that case. Finally, the tool wear states are divided into four categories whose scopes are shown in Table 2.

### 3.4. Data Preparation

**Figure 5.**
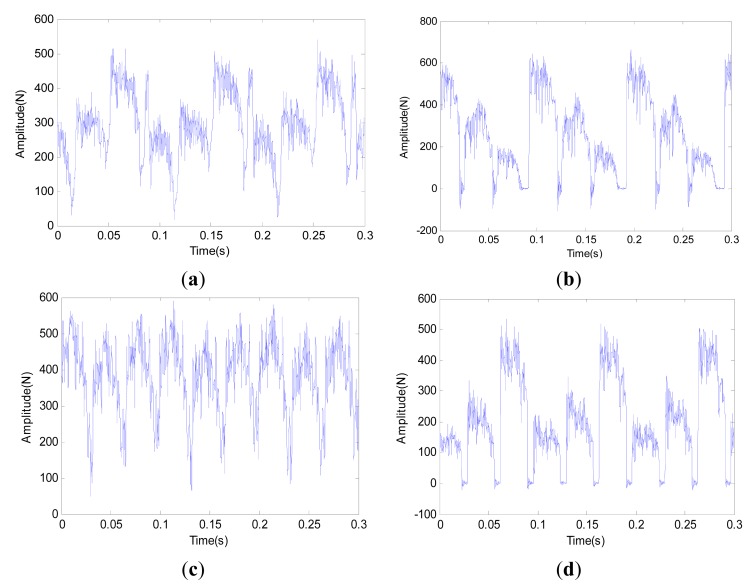
Force waveforms for different tool wear categories. (**a**) New tool; (**b**) Initial wear; (**c**) Middle wear; (**d**) Severe wear.

**Figure 6.**
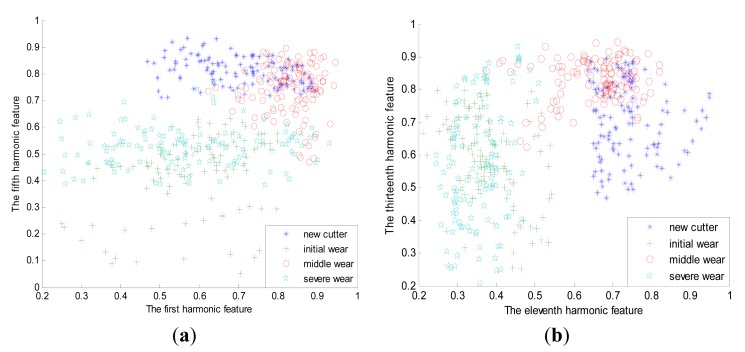
Spatial distributions of feature vectors for different tool wear categories. (**a**) The first and fifth harmonic feature; (**b**) The eleventh and thirteenth harmonic feature.

Within each tool wear category, the dynamic force signal is split into 240 segments with the length of 4096 points so as to cover the dynamic characteristic of all inserts. Therefore the number of the samples in the whole dataset is 960. Figure 5 depicts the waveforms of typical force signals under four kinds of tool wear states. It can be seen that the periodicity of the sensory signal is obvious. Moreover, noisy interference also exists, which makes it hard to be used directly as feature vector. To depict the dynamic characteristics of the force signal, the first 16 order harmonic features are extracted for each sample in the dataset by using Equations (4) and (5). Moreover, to improve relevance and reduce redundancy simultaneously, the mRMR algorithm is further adopted to select the most prominent features based on the whole dataset. Finally, the first, second, fifth, eleventh and thirteenth harmonic features are selected. The spatial distribution of these feature vectors is illustrated in Figure 6. It can be seen that these feature vectors distribute dispersedly and the shape is irregular, which casts higher demand on the construction of the classifier. To analyze the accuracy and stability of the classifier, the whole dataset is divided into two parts equally. One part is used to train the ensemble learning classifier and the other is to test the accuracy of the built classifier. Moreover, the training and testing process of the classifier is repeated 10 times and both the average accuracy and deviation are calculated simultaneously to accurately evaluate the classifier.

### 3.5. Analysis and Discussion

#### 3.5.1. Comparison with Single Classifiers

**Figure 7.**
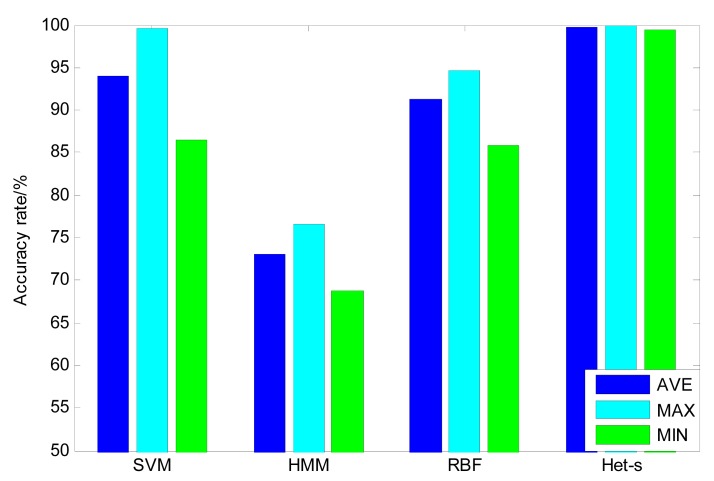
Comparison of single classifier with heterogeneous ensemble classifier (Het-s—heterogeneous stacking; AVE—averaging accuracy; MAX—maximum accuracy; Min—minimum accuracy).

Based on the above training samples, the heterogeneous ensemble learning classifier with stacking strategy is constructed. The classification results for the test samples are given in Figure 7. It can be seen that the maximum accuracy of the heterogeneous ensemble learning classifier is 100% and the minimum accuracy is 99.38%. The average accuracy can reach 99.79%. For comparison purposes, the single classifiers based on SVM, HMM and RBF, respectively, are also built and the classification results are given in Figure 7. It can be seen that SVM can achieve the highest accuracy among these single classifiers, which amounts to 93.94%, while the average accuracy of HMM is lowest, which is 73.1%. In order to further illustrate the stability of the classifier, the standard deviation of single classifier and heterogeneous ensemble learning classifier are computed and listed in Table 3. It can be found that HMM has the best stability among the three single classifiers while the stability of the SVM classifier is the worst. In contrast, the standard deviation of heterogeneous ensemble learning model is only 0.22%, which is far less than that of the HMM classifier. Therefore, it can be concluded that the heterogeneous ensemble can lower the risk of wrong classification so as to improve the stability and accuracy of the classifier [29].

#### 3.5.2. Comparison with Homogeneous Ensemble Learning

**Figure 8.**
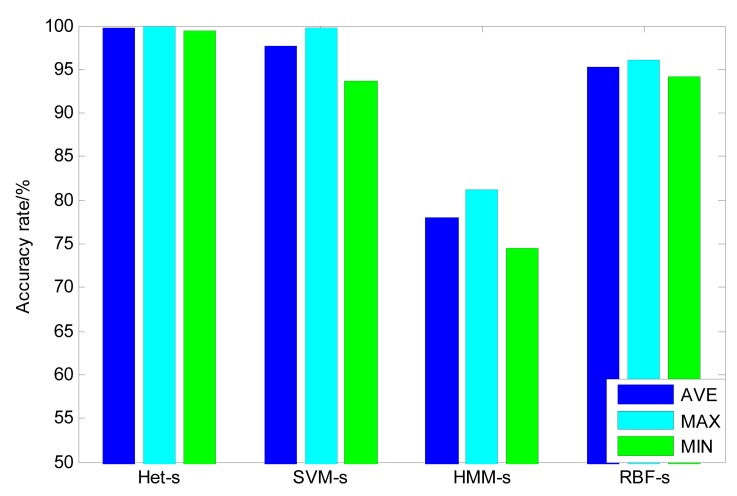
Comparison of heterogeneous with homogeneous ensemble classifier (Het-s—heterogeneous ensemble with stacking, SVM-s—SVM homogeneous ensemble with stacking, HMM-s—HMM homogeneous ensemble with stacking; RBF-s—RBF homogeneous ensemble with stacking).

In this section, heterogeneous ensemble learning is further compared with the homogeneous ensemble learning based on the same stacking strategy. Different from the heterogeneous ensemble, homogeneous ensemble learning uses the same kind of model as base classifiers [30]. However, their initialization parameters and weight values are totally different from each other. Therefore, in comparison with single classifiers, some wrong results within each single model are revised by combining different outputs and the final classification accuracy can be improved to some extent [31]. In this paper, based on SVM, RBF and HMM, respectively, three homogeneous ensemble learning classifiers are constructed. The comparison between the heterogeneous and homogeneous classifiers is shown in Figure 8. It can be seen that, among the three homogeneous ensemble classifiers, the SVM-based model has the best average and maximum accuracy, which are 97.73% and 99.79% respectively. What's more, the RBF-based model has the best minimum accuracy, which is 94.17%. While for the heterogeneous ensemble classifier, the average accuracy can achieve 99.79%, the maximum accuracy is 100% and the minimum accuracy is 99.38%. The deviations of these homogeneous classifiers are given in Table 3. It is shown that RBF has the best stability among these homogeneous classifiers and SVM is the worst. In contrast, the standard deviation of classifier is only 0.22%, which is far lower than that of homogeneous ensemble classifiers. These results prove that the heterogeneous ensemble has better accuracy and stability than the homogeneous ensemble. One reason is that the lower correlation between the errors of each heterogeneous base classifier reduces the ensemble error of the final ensemble learning classifier. Another reason for these results is that the diversity of the heterogeneous ensemble learning is larger than that of the homogeneous ensemble, which makes the SVM stacking in the second levels more likely pick up the most useful support vectors so as to depict the final decision boundary more accurately if the accuracy of the base classifiers is high enough [30].

#### 3.5.3. Comparison with Majority Voting

**Figure 9.**
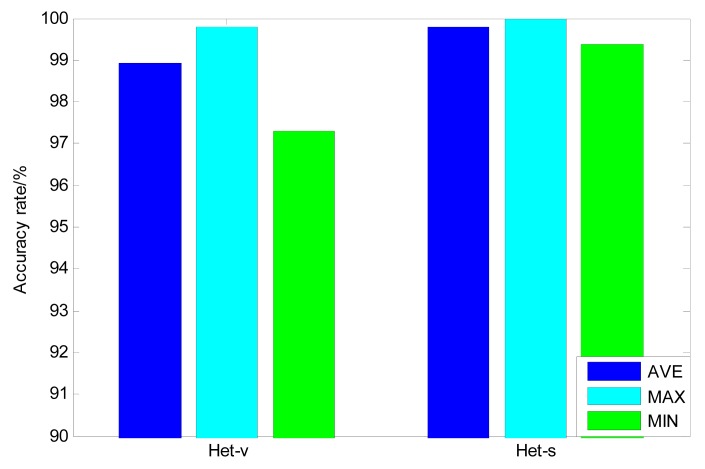
Comparison of major voting with stacking strategy for heterogeneous ensemble classifier (Het-v—heterogeneous ensemble with majority voting and Het-s—heterogeneous ensemble with stacking).

In order to show the advantage of SVM-based stacking strategy, another kind of ensemble strategy-majority voting is also adopted to construct a heterogeneous ensemble learning classifier based on the above three base classifiers. Different from the SVM stacking strategy, majority voting sums the predictions of every base classifier and picks the most popular class [21]. The classification results of the majority voting are illustrated in Figure 9 and the deviations are listed in Table 3. The comparison with SVM-based stacking demonstrates that the average accuracy and deviation of the SVM-based stacking exceeds that of the majority voting algorithm, which proves that strong nonlinear mapping ability of SVM-based stacking is more effective to rectify the prediction errors of certain base classifiers, which guarantees that the final classification results is more accurate [21].

## 4. Conclusions

A tool condition monitoring system is built based on the heterogeneous ensemble learning classifier. In this system, three kinds of different models, *i.e.*, SVM, RBF and HMM are selected as the base classifiers and a stacking strategy is used to integrate the outputs of these base classifiers and judge the final tool wear category. In order to verify the effectiveness of the proposed method, titanium alloy milling experiments were carried out and signals from the force sensor were collected to depict the dynamic characteristics of the machining process. The harmonic feature vectors are extracted and further selected based on the mRMR algorithm to build the heterogeneous ensemble learning classifier. Moreover, a homogeneous ensemble learning and majority voting strategy are also adopted to make a comparison. The analysis and comparison results show that the average accuracy of the heterogeneous learning classifier is the highest, while its standard deviation is the lowest among these classifiers. These results testify to the effectiveness of the proposed heterogeneous ensemble classifier in the field of tool condition monitoring.

## Figures and Tables

**Table 1.** List of cutting parameters.

**Cutting Speed**	**Feed Rate**	**Cutting Width**	**Cutting Depth**	**Cutter Diameter**	**Number of Tooth**
597 rpm	0.1 mm/rev	18 mm	1 mm	30 mm	3

**Table 2.** Tool wear categories and its corresponding wear scope.

**Tool Wear Category**	**New Tool**	**Initial Wear**	**Middle Wear**	**Severe Wear**
Wear value (mm)	<0.1	0.1–0.2	0.2–0.35	>0.35

**Table 3.** Standard deviation of accuracy for different classifiers.

**Classifier**	**Single Classifier**			**Heterogeneous Ensemble**		**Homogeneous Ensemble**		
		
	**SVM**	**HMM**	**RBF**	**SVM stacking**	**Majority Voting**	**SVM**	**HMM**	**RBF**
Deviation (%)	4.77	2.39	4.09	0.22	0.96	2.3	2.04	0.81
